# Training Schools in Relation to Size

**Published:** 1908-10-24

**Authors:** 


### Nursing Administration.

### TRAINING SCHOOLS IN RELATION TO SIZE.

The advantages of large training schools and the drawbacks of small ones are sufficiently notorious. So much so, indeed, that they have penetrated through to the public, and the small establishments begin to experience a growing shortage of probationers willing to submit themselves for training. Large and small are relative expressions. Training schools of the first magnitude containing a nursing staff of 100 and upwards number only 14, of which eight are in London, and four in Scotland. The number of beds in these major institutions is with two exceptions over 300, and in each case a medical school is attached. Several other provincial infirmaries, however, fall properly into this group, though numerically somewhat below the rest. Next in order come a group of training schools containing 100 or more beds, with a nursing staff of 25 or more, and this group falls into two divisions, according to the existence or not of a medical school. Lastly, comes a group of distinctly small hospitals with less than 100 beds, and a nursing staff of under 25. This group has been divided into two again by the regulations of the Royal British Nurses' Association, which recognises hospitals having 40 beds and upwards as qualified to train nurses, and excludes the rest, whereas other advocates for registration are pressing for a rule excluding all hospitals below 50 beds from this privilege. All these divisions are somewhat arbitrary. Wherever the line is drawn there is always a group of hospitals below it which partake more of the character of the upper division than of their own. In other words, the factors which mark out a hospital as belonging to a special group or division are many in number, and size is but one of them. It is interesting, in view of recent discussion, to consider what size can do, and how far it can be claimed as the principal matter in determining the choice of a training school.

In the first place size undoubtedly lends prestige, and the nurse retains some of the distinction attaching to her training school as an asset through life. Again size brings emulation, and that induces keenness in work and a desire to excel. The consciousness of taking part in a great organisation, and the opportunity of gaining distinction in it are valuable aids through all the drudgery part of nursing. More important still is the fact that the best medical and surgical work is afforded in large establishments. Nurses have the opportunity of seeing a quick succession of acute cases in the wards of the larger hospitals. They become familiar with disease under its more forcible manifestations, and develop a readiness in dealing with emergencies only to be obtained by constant contact with complicated cases. They get a chance of being well grounded in special work, such as is to be met in the ophthalmic or gynæcological wards. They hear stimulating lectures. They see their work continually developing before them, so that even at the end of three or four years' service there are desirable forms of experience still to be gained, and posts of an influential character to be aimed at. This maintains almost without effort an excellent spirit among the nurses in big institutions. Lastly, the large institution can offer, as a rule, better accommodation to its nurses, more cheerful opportunities for recreation and a more congenial community life.

Yet, with all these things in their favour, it is by no means certain that the larger training schools turn out better instructed nurses than their less well-equipped sisters. Nurses who are justly proud of their world-famed schools are loudly declaring that no other training is worthy of the name. And yet there are those who know who can testify on the other side. Some who have themselves been trained and have held responsible posts in large hospitals, and have passed subsequently to the superintendence of smaller institutions have much to urge in favour of the smaller hospitals as training schools. Freely admitting, and, indeed, glorying in the distinction of their own *alma mater*, these matrons are setting themselves to work valiantly to produce similar effects with different instruments. The keenness which is not engendered automatically in a little hospital they aim at inspiring through a sense of duty. What is lost in the absence of a stimulating pressure of work, they endeavour to supply by the exercise of personal influence in moulding the character of the probationer. In the actual work of the wards they contend that though there may be less variety there is more certainty that each nurse gets her full share of such medical and surgical experience as the hospital affords. They may not often see uncommon phases of disease, but in the mixed general wards of the little hospital the nurse is thoroughly grounded in regard to cases which she may well happen never to see at all during the time spent in a training school too large to give all probationers, even in four years; work in every department. True the nurse will have fewer acute cases falling under her observation, but she will be nursing them on into convalescence, and will have opportunities for observation of disease in all its stages, approximating far more nearly to what will be expected of her in private work than are possible under more severe pressure on the beds. Lastly, what is wanting in luxury may be easily made up in solid comfort. There is nothing more delightful than the happy tone of intercourse which prevails in some small hospitals where the matron is much with her nurses, and lends a zest to every trifle by her own interest in the life. It is unjust to disparage the good work which is being done in countless establishments whose beds fall below even the lowest estimate of the number a training school should possess. The country needs all the nurses it can get trained under good conditions, even when the training is carried out at some disadvantage as regards variety of experience.

